# Fertility of Crossbred Dairy Cattle After Progesterone‐Supplemented Co‐Synch in Southwestern Ethiopia

**DOI:** 10.1155/vmi/1243812

**Published:** 2026-02-15

**Authors:** Aregaw Abera Dodicho, Sileshi Tadesse Gebeyehu, Zelalem Yilma Kidane

**Affiliations:** ^1^ Department of Animal Science, College of Agriculture and Veterinary Medicine, Jimma University, PO Box 307, Jimma, Ethiopia, ju.edu.et; ^2^ The Korea International Cooperation Agency (KOICA), P.O. Box 5652, Addis Ababa, Ethiopia; ^3^ Land O’Lakes Venture37, P.O. Box: 5689-ILRI, Addis Ababa, Ethiopia

**Keywords:** crossbred, estrus, pregnancy rate, progesterone

## Abstract

In Ethiopia, the reproductive efficiency of dairy cattle utilizing artificial insemination (AI) is low, and implementing estrus and ovulation synchronization protocols could enhance the efficiency. Therefore, this study was designed to evaluate the effect of progesterone (P4) supplementation during Co‐Synch on estrus response (ER) and pregnancy per AI (P/AI) in crossbred dairy cattle breed in Southwestern Ethiopia. In the trial, heifers and cows (*n* = 120) were enrolled in the study. All animals received 100 μg of gonadotropin‐releasing hormone (GnRH) and had a progesterone‐releasing intravaginal device (PRID) inserted intravaginally on Day 0. On Day 7, they received 25 mg of prostaglandin (PGF2*α*), followed by the removal of the PRID. On Day 9, animals received 100 μg of GnRH concurrent with insemination. Estrus signs were monitored to assess heat response during morning and evening for at least 30 min at approximately 12‐h intervals from PRID removal on Days 7 through 9. Trans‐rectal ultrasonography was used to diagnose pregnancy 60 days after AI. The P/AI was recorded as the total number of pregnant females from the total number of animals involved in the synchronization program. A logistic regression analysis was performed to test the effectiveness of the treatment on ER and PR. 80% of animals showed ER to the treatment. A greater percentage of primiparous cows (93.33%) responded to the treatment, followed by heifers (83.33%), while the lowest response was seen in cows with a parity of 3 (66.67%). A total of 59.17% of the overall P/AI was achieved. The P/AI was affected by parity; a higher P/AI was obtained from the primiparous group. Statistically, no bull or BCS effect on P/AI was observed in the current study. Supplementation of P4 improved pregnancy outcomes, surpassing the national average, suggesting the protocol’s success in synchronizing estrus and ovulation among crossbred cows and heifers.

## 1. Introduction

Ethiopia has a sizable livestock population, a climate conducive to improving high‐yield dairy cattle breeds, and regions with reduced animal disease pressures, all of which contribute to the country’s considerable potential for dairy development [1, 2]. Among livestock species, cattle contribute significantly to farmers’ livelihoods as a primary source of wealth, providing nutrition, manure, transport, and draught power, and are often used to pay as bride wealth [3, 4]. The country has about 70.29 million cattle [1, 5]. Most of these animals belong to Indigenous breeds distributed across diverse topographic and climatic conditions, with less than 2% of crossbred dairy cattle [6, 7]. Despite possessing such a huge potential, the sector has not progressed as anticipated due to a limited number of crossbred dairy cows, shortages in both the quantity and quality of feed, inadequate infrastructure, and subpar market services [8–10]. Furthermore, the challenges are compounded by the unsatisfactory reproductive performance of dairy cattle and their breeding management. Repeat‐breeding, long inter breeding intervals, anestrus, poor estrus detection practices, higher numbers of services per conception, reduced conception rate, and long days open are among the major constraints hindering the reproductive efficiency of dairy cattle breeding across the country [11–13]. To overcome such challenges, controlled breeding programs have become standard practices in the reproductive management of dairy herds [14].

Protocols for synchronizing the estrous cycle and timed artificial insemination (TAI) have been implemented worldwide to control reproduction systematically in dairy herds [15]. Several synchronization strategies have been developed to improve reproductive efficiency, optimize labor and management, and enhance herd genetics and profitability in the dairy industry. The most commonly practiced are 5‐day CO‐Synch [16–18], 7‐Day CO‐synch [19], Ovsynch [20–23], Presynch‐Ovsynch [24, 25], and Double‐Ovsynch [25, 26]. The 7‐day CO‐Synch protocol offers advantages over others primarily in simplicity and reduced handling compared to more intensive protocols, making it a common choice for routine use. Additionally, its effectiveness is enhanced with the addition of a progesterone (P4)‐releasing device [27, 28]. As a result, estrus and ovulation have been induced using intravaginal devices that release P4, in conjunction with gonadotropin‐releasing hormone (GnRH) and prostaglandin F2*α* (PGF2*α*) [29, 30]. As the name indicates, the 7‐Day CO‐Synch is the standard protocol, involving a 7‐day interval between the first GnRH injection and the PGF2*α* injection in dairy cattle breeding [31–33]. Reduced pregnancy rate (PR), reduced embryo development, and embryonic losses have been associated with low or abnormal P4 concentrations after AI in dairy cattle [34–37]. To alleviate these problems and also to improve synchronization and conception rates, the use of a P4 releasing device like a progesterone‐releasing intravaginal device (PRID) insert (CO‐Synch + PRID) was included in the protocol [38, 39]. As a result, improved conception rates by enhancing synchronization and embryo survival [18, 38, 40–42] have been reported. Furthermore, a higher proportion of dairy heifers treated with a P4 supplementation and PGF2*α* showed signs of estrus compared with applying only PGF2*α*. Despite the protocol having such a positive effect in improving reproductive efficiencies, the positive effect of supplementation with P4 was not consistent across all locations. Lamb et al. [43] reported that cows in Missouri (62%) and Kansas (60%) had greater PRs than those in Illinois (47%) and Minnesota (44%). In another study, Lamb et al. [44] also recorded PRs of 39%–74% among different locations in heifers using a similar approach. Additionally, although these protocols are widely implemented across different parts of the world, the effectiveness of field CO‐Synch programs is less known in tropical climates like Ethiopia. A clear understanding of overall PRs from PRID‐based fixed‐time artificial insemination (FTAI) programs like 7‐day CO‐Synch is not available. Therefore, the current study was devised to evaluate the effect of P4 supplementation in the CO‐Synch protocol on estrus response (ER) and PR in crossbred dairy cattle under an urban dairy production system in Southwestern Ethiopia.

## 2. Materials and Methods

### 2.1. Description of the Study Area

**FIGURE 1:**
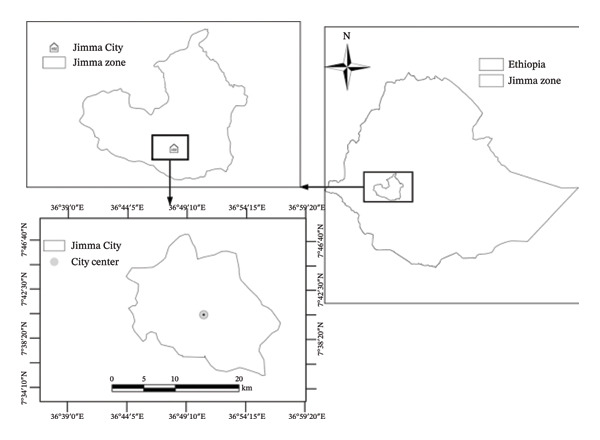
Study area, Jimma town, located in Jimma zone of Ethiopia: Source [45].

The research was carried out in Jimma town (see Figure 1), situated in the Oromia Regional State, approximately 352 km southwest of the capital. The area receives annual rainfall between 1400 and 1900 mm, with average maximum temperatures ranging from 25°C to 30 °C and minimum temperatures between 7°C and 12 °C. The area is primarily recognized for its production of coffee, crops, and livestock.

### 2.2. Study Animals and Their General Management

A total of 20 dairy farms were purposively selected based on uniformity in overall management practices in the study area. Most farms were small (one to five cattle) and medium (6–20 cattle) scale farms. All the farms practiced an intensive farming system offering their animals a variety of feedstuffs, including local concentrate feed, hay, and straw, with ad libitum water. A total of 120 animals, which met the trial criteria, which included factors such as overall health, body condition, parity, and normality of the reproductive system, were enrolled in the study. All animals enrolled in the study were crossbred dairy cattle (Zebu [native breed to Ethiopia] X Holstein Friesian) with the blood level of the Holstein Friesian ranging from 50% to 75%. The animals’ body condition score (BCS) ranged from 2.5 to 4.0 on a 1 to 5 BCS scale measurement. Regarding parity, an equal number of animals were included from heifers, parity 1, 2, and 3. The Jimma University College of Agriculture and Veterinary Medicine Animal Care and Use Committee approved animal‐related procedures (College of Agriculture and Veterinary Medicine ethical committee, RP/March 7/2024).

### 2.3. Study Design

**FIGURE 2:**
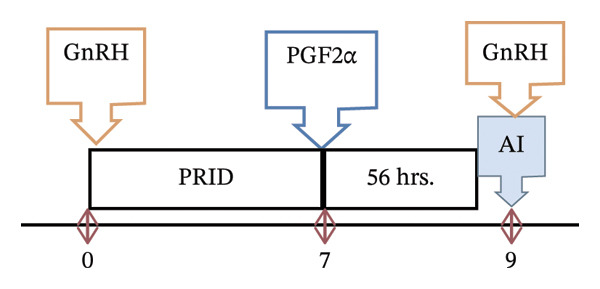
Schematic representation of the study design.

Before the synchronization, the absence of pregnancy in the animals was confirmed using ultrasonography. All animals (cows and heifers, *n* = 120) in the study protocol received a PRID (PRID DELTA, containing 1.55 g of P4) plus GnRH (100 μg i.m.) upon PRID insertion on Day 0 (D0). On Day 7 (D7), all animals were injected with PGF_2α_ (25 mg dinoprost i.m.; Enzaprost) on PRID removal. Then, after 56 h, the second round of GnRH was administered to all animals along with an AI application. Pregnancy diagnosis was carried out 60 days after the insemination (see Figure 2).

### 2.4. Estrus Response and Pregnancy Rate

Although all animals were inseminated at a fixed time, observations for behavioral estrus were conducted for 30 min at approximately 12‐h intervals from PRID removal to determine estrus synchrony. Trans‐rectal ultrasonography was used to diagnose pregnancy 60 days after artificial insemination. Then, the PR was recorded as the total number of pregnant females from the total number of females in the synchronization program.

### 2.5. Statistical Analysis

The effect of parity (classified as heifers, primiparous, parity 2 and 3), BCS (2.5, 3, 3.5, and 4), and bull (1, 2, and 3) on estrus response and pregnancy rates was assessed using logistic regression. The reference category for comparing odds ratios (ORs) was heifers, BCS 2.5, and bull 1 for parity, BCS, and bull, respectively. Results are illustrated as percentages, estimated OR, and 95% confidence interval. Statistical differences were considered significant when *P* < 0.05. Statistical analyses were conducted using SAS 9.4 (SAS Inst. Inc., Cary, NC).

## 3. Results and Discussion

### 3.1. Estrus Response

The expression of estrus can be affected by various factors, including genetic traits, the number of days postpartum, milk production, overall health, weather conditions, stress, hormonal treatment, bull presence, housing, and herd size [46–48]. Out of 120 animals (heifers and cows) enrolled in the trial, 96 (80%) responded to the treatment by showing different signs of estrus (Table 1). ER across parities did not differ (*P* > 0.05) and was 83.33% (25/30) in heifers, 93.33% (28/30) in primiparous, 76.67% (23/30) in parity 2%, and 66.67% (20/30) in parity 3 cows. Even though there is no statistical difference among the parity groups, the result indicates that animals in the primiparous group were 1.1062 times more likely to respond to the treatment compared to the reference group while the animals in parity 3 were 0.83 times less likely to respond to the treatment (OR = −0.8397; 95% CI: 0.118–1.360) as compared to their counterparts in the reference groups. This indicates that the efficacy of the treatment regime is better in heifers and primiparous cows than that in multiparous cows in the study. Potential factors contributing to improved fertility traits like ER in primiparous cows may include a lower likelihood of experiencing metabolic disorders during early lactation [49, 50] compared to multiparous cows. Piñeyrúa et al. [51] also noted the effects of parity in the metabolic and hormonal profiles of the animals, where a better energy balance was observed for the primiparous cows than for the multiparous cows. It also could be associated with the animals’ pretreatment cyclic status, which can affect the number of cows detected in estrus [52]. Overall, the results from the current study are in line with the results reported by previous researchers. Romano and Fahning [53] obtained a 79.5% ER in CIDR‐7 groups. Similarly, Siregar et al. [54] also reported an 80.0% ER in animals synchronized using CIDR inserted into the vagina for 7 days and followed by injection of PGF2*α* 5 mL intramuscularly on Day 6, while Lucy et al. [52] recorded 84%. Bonacker et al. [31] also obtained a comparable ER (76%) in the 7‐day CO‐Synch CIDR protocol. A higher ER (100%) was achieved in crossbred dairy cows using both single and double PGF2*α* injection protocols under smallholder farming conditions [55]. This approach is simpler to implement and more cost‐effective than the protocol used in the current study. Similarly, a higher ER, such as 91.01% in the local Boran breed [56], 94.7% in the local breed [57], and 97.7% involving both local and crossbred cows and heifers [58], was achieved using PGF2*α* injection. Of course, these differences might not be due to the treatment protocols implemented. Animal management systems implemented during the study period, such as feeding in different studies or breed differences in animals involved in other studies, could have affected the physiological responses to synchronization [42, 52]. A contrasting result was also reported by Bonato et al. [59], where a lower ER was recorded in the primiparous (61.36%) group than in the heifers (76.39%) and multiparous (75.69%) groups. The disagreement might be due to the different animal management systems implemented in the two studies. The authors attributed the lower ER in primiparous cows to the metabolic challenges they experienced, as they were raised on a pasture‐based system with only mineral supplementation.

**TABLE 1:** Effect of a progesterone‐releasing intravaginal device (PRID) during a 7‐day CO‐Synch protocol on estrus response in crossbred dairy cattle under urban dairy production system.

Characteristics	Estrus response			95% confidence interval	*P* value
	Animals responded (%)	Animals not responded (%)	Odds ratio		
Parity					0.1011
Heifers	25/30 (83.33)	5/30 (16.67)	Heifers (reference)		
Primiparous	28/30 (93.33)	2/30 (6.67)	1.1062	0.498–15.734	0.0568
Parity 2	23/30 (76.67)	7/30 (23.33)	−0.3432	0.183–2.363	0.3948
Parity 3	20/30 (66.67)	10/30 (33.33)	−0.8397	0.118–1.360	0.0272
Total	96/120 (80)	24/120 (20)			
BCS					**0.3119**
2.5	14/17 (82.35)	3/17 (17.65)	BCS 2.5 (reference)		
3.0	30/35 (85.71)	5/35 (14.29)	−0.4292	0.389–4.245	0.2958
3.5	41/48 (85.42)	7/48 (14.58)	0.2399	0.721–8.743	0.5775
4.0	11/20 (55)	9/20 (45)	0.8698	0.492–45.126	0.2796
Total	96/120 (80)	24 (20)			

*Note:* Bold value indicates probability value of test statistics.

### 3.2. Pregnancy Outcomes

The overall pregnancy per AI (P/AI) was 59.17% (71/120) (Table 2). The P/AI was influenced by parity (*P* = 0.0012). As the result indicates, animals in primiparous groups were 0.9601 times more likely to become pregnant than the reference group (*P* = 0.0118) while the animals in parity 3 were 1.1193 times less likely to be pregnant using the same protocol than heifers (OR = −1.1193; 95% CI: 0.060–0.551). Although not statistically significant, animals in their second parity were 0.4262 times less likely to be pregnant than those in the reference groups. Based on these findings, the treatment regimen appears to be more effective in heifers and primiparous cows than in multiparous cows in this study. The higher P/AI observed in heifers and primiparous cows relative to multiparous cows may be due to various factors. The P/AI results followed a similar pattern to the ER, with heifers and primiparous cows demonstrating a better ER before TAI (Table 1). The impact of expression of estrus on the overall success of a pregnancy outcome has been well documented in the literature. Ferraz et al. [60] evaluated the reproductive performance of Nellore cows involving heifers, primiparous, and multiparous cows submitted to the 7dEB + P4 system and recorded that greater P/AI in cows detected in estrus than in cows not detected in estrus. Similar results were reported by other researchers. Rodrigues et al. [61] noted that cows with low or no estrus expression present lower P/AI in TAI protocols. A similar study by Sá Filho et al. [62] also observed a positive effect of estrus on P/AI in Nelore cows with protocols using EC or GnRH and estrus was evaluated with tail chalk. Colazo et al. [63] also observed a better ER in heifers and primiparous cows and noted that estrus expression before TAI affected P/AI at both 33 and 61 days post‐TAI. Similarly, Zwiefelhofer et al. [28] reported an overall increase in P/AI in cattle exhibiting estrus in cattle given a PRID. Additionally, Nogueira et al. [64] reported the influences of estrus expression on PRs in *B. indicus* cows subjected to TAI with P4E2‐based protocol. They found that the PR of animals that show estrus is 20.91% higher than that of cows that do not express estrus. Cows that fail to exhibit estrus during TAI often have poor P/AI rates, likely because they do not respond properly to the synchronization protocol. Additionally, these cows typically lack a dominant follicle of sufficient size to produce the estrogen needed for estrus expression [59, 65]. Furthermore, the association between ER and P/AI in the current study could be attributed to physiological factors like differences in the cycling status of the animals preceding synchronization. In the current study, animals were allocated to the treatment at random stages of the estrous cycle. Following this, Demissie et al. [66] reported a greater conception rate in cycling heifers at day zero than in noncycling. Correspondingly, Ramoun et al. [67] also reported a beneficial effect of supplemental P4 via PRID insertion on fertility response in cyclic cows compared to acyclic cows using a 0–7 days Ovsynch protocol. It could also be associated with the cyclic and P4 status before synchronization. Hill et al. [68] observed less P/AI in anestrous cows with low P4 status at the onset of the TAI program than in cycling cows with low P4 status, as well as in anestrous cows with high P4 status. Additionally, Stevenson et al. [69] also noted that cycling status preceding synchronization and cycling status × parity as significant sources of variation in pregnancy outcomes. This could be attributed to differences in follicular dynamics between cyclic and noncyclic [70], since estrous cyclicity depends on ovarian follicular development, and the response to estrous synchronization also depends on ovarian follicular development [71]. It could also be due to differences in the presence of functional corpus luteum (CL) at PRID insertion on Day 0 among the experimental animals, which could be a potential source of variation in ovulation rate and consequently in conception rate [72]. In agreement with this, Rivera et al. [39] and Colazo et al. [63] reported a greater P/AI in cows with an active CL than in cows without a CL after administering exogenous P4, and Rivera et al. [39] pointed out that administering exogenous P4 without an active CL reduces fertility in cattle due to the formation of persistent follicles. Other factors, such as concentrations of P4 before the synchronization program, metabolic status of the animals, differences in days postpartum in multiparous cows, and farm‐specific characteristics, could also be a source of the observed difference in pregnancy outcomes in the current study. In this study, significant differences were observed in P/AI among BCS groups. Surprisingly, animals from the BCS of 2.5 had better PRs than their counterparts from the 3.0 and 3.5 categories. The results show that animals from the BCS of 3.0 were 0.7553 less likely to become pregnant than the reference group. Increased P/AI in the animals from lower BCS compared to higher BCS in this study corroborates the findings of Randi et al. [73], who also detected 15% higher P/AI in low BCS animals compared to those in moderate to high BCS in cows synchronized with the standard CO‐Synch + PRID protocol. The authors justified that it could be due to cows gaining BCSs during the breeding season, as pasture production quality progressively increases during the breeding season. Hence, the better P/AI in animals with lower BCS in the current study could also be due to animals gaining BCSs during the breeding season. In contrast to animals from lower BCS, animals from BCS 4.0 were 1.6425 times more likely to become pregnant as compared to the reference group. This higher P/AI in animals from BCS four is in line with the fact that animals with adequate body conditions have better reproductive performance (fertility) [74, 75]. Other researchers also reported greater P/AI from cows with a greater BCS index [76, 77]. In line with this fact, the result of the meta‐analyses conducted by Bedere et al. [78] shows that the conception rate to first insemination was increased by 38.2% units for each additional BCS at AI. In contrast, low BCS has been identified as a risk factor for a reduction in overall reproductive performance in dairy cattle. Poor fertility in animals from low BCS is particularly associated with delayed commencement of luteal activity and embryo survival [79, 80]. Additionally, while we cannot give a formal explanation, there could also be farm‐specific and animal effects on observed differences in the P/AI in the current study. We speculate that certain farm‐specific factors (such as housing and feeding/nutrition) and animal factors like days postpartum at treatment initiation may have influenced the pregnancy outcomes. No bull effect on P/AI was observed in the current study.

**TABLE 2:** Effect of a progesterone‐releasing intravaginal device (PRID) during a 7‐day CO‐Synch protocol on pregnancy outcomes in crossbred dairy cattle under an urban dairy production system.

Characteristics	Pregnancy rate			95% confidence interval	*P* value
	Pregnant (%)	Nonpregnant (%)	Odds ratio		
Parity					0.0012
Heifers	22 (73.33)	8 (26.67)	Heifers (reference)		
Primiparous	24/30 (80)	6 (20)	0.9601	0.435–4.860	0.0118
Parity two	15/30 (50)	15 (50)	−0.4262	0.123–1.071	0.1948
Parity three	10/30 (33.33)	20 (66.67)	−1.1193	0.060–0.551	0.0010
Total	71 (59.17)	49 (40.83)			
BCS					**0.1608**
2.5	11/17 64.71)	6/17 (35.29)	BCS 2.5 (reference)		
3.0	20/35 (57.14)	15/35 (42.86)	−0.7553	0.279–2.402	0.0454
3.5	29/48 (60.42)	19/48 (39.58)	−0.3325	0.435–3.583	0.3674
4.0	11/20 (55)	9/20 (45)	1.6425	0.969–83.579	0.0397
Total	71/120 (59.17)	49/120 (40.83)			
Bull					**0.1272**
1	28/40 (70)	12/40 (30)	Bull 1 (reference)		
2	19/40 (47.5)	21/40 (52.5)	−0.4843	0.155–0.971	0.0659
3	24/40 (60)	16/40 (40)	0.0213	0.255–1.623	0.9362

*Note:* Bold values indicate probability values of test statistics.

## 4. Conclusion

This study aimed to evaluate the effect of P4 supplementation via PRID in a 7‐day Co‐Synch protocol on ER and P/AI involving crossbred dairy cows and heifers in southwestern Ethiopia as a trial before a large operation. In conclusion, heifers and primiparous females had a higher rate of estrus as well as P/AI. Furthermore, our findings demonstrated acceptable PR results in the 7‐day P4‐based Co‐Synch protocol for crossbred dairy cattle in the study area. Hence, the protocol can enhance the fertility of dairy cattle in the study area and other regions if implemented on a large scale after further evaluating P/AI, considering the cyclic status and serum P4 level before and during the synchronization period.

## Author Contributions

Aregaw Abera Dodicho: conceptualization, data curation, formal analysis, investigation, methodology, supervision, writing–original draft, and writing–review and editing. Sileshi Tadesse Gebeyehu: conceptualization, data curation, formal analysis, investigation, methodology, writing–original draft, and writing–review and editing. Zelalem Yilma Kidane: conceptualization, data curation, formal analysis, investigation, methodology, supervision, writing–original draft, and writing–review and editing.

## Funding

No funding was received for the study.

## Conflicts of Interest

The authors declare no conflicts of interest.

## Data Availability

The authors confirm that the data supporting this study’s findings can be obtained from the principal author upon reasonable request.
